# Supplementary material for: Methylation of recombinant mononucleosomes by DNMT3A demonstrates efficient linker DNA methylation and a role of H3K36me3
Source: Commun Biol. 2022 Mar 2;5:192. doi: 10.1038/s42003-022-03119-z (PMC8891314; doi:10.1038/s42003-022-03119-z)
Supplement: Supplementary file 2 — Supplemental Information [file 42003_2022_3119_MOESM2_ESM.pdf]

Methylation of recombinant mononucleosomes by DNMT3A demonstrates efficient linker DNA methylation and a role of H3K36me3

Alexander Bröhm, Tabea Schoch, Michael Dukatz, Nora Graf, Franziska Dorscht, Evelin Mantai, Sabrina Adam, Pavel Bashtrykov & Albert Jeltsch*

SUPPLEMENTAL INFORMATION

Supplemental Figures

Supplemental Figure 1: Sequence alignment of human and mouse DNMT3A, DNMT3B, DNMT3B3 and DNMT3L.

Supplemental Figure 2: Quality control of the modified histones and octamer purification.

Supplemental Figure 3: Controls related to nucleosome reconstitution and methylation.

Supplemental Figure 4: Additional data related to nucleosome methylation.

Supplemental Figure 5: Additional data related to the purification of DNMT3AC/3B3C heterotetramers.

Supplemental Figure 6: Fluorescence spectroscopy experiments investigating the interaction of the H3-tail with the linker DNA.

Supplemental Figure 7: Uncropped versions of gel images shown in the main figures.

Supplemental Tables

Supplemental Table 1: List of nucleosome and DNA methylation experiments.

Supplemental Figures

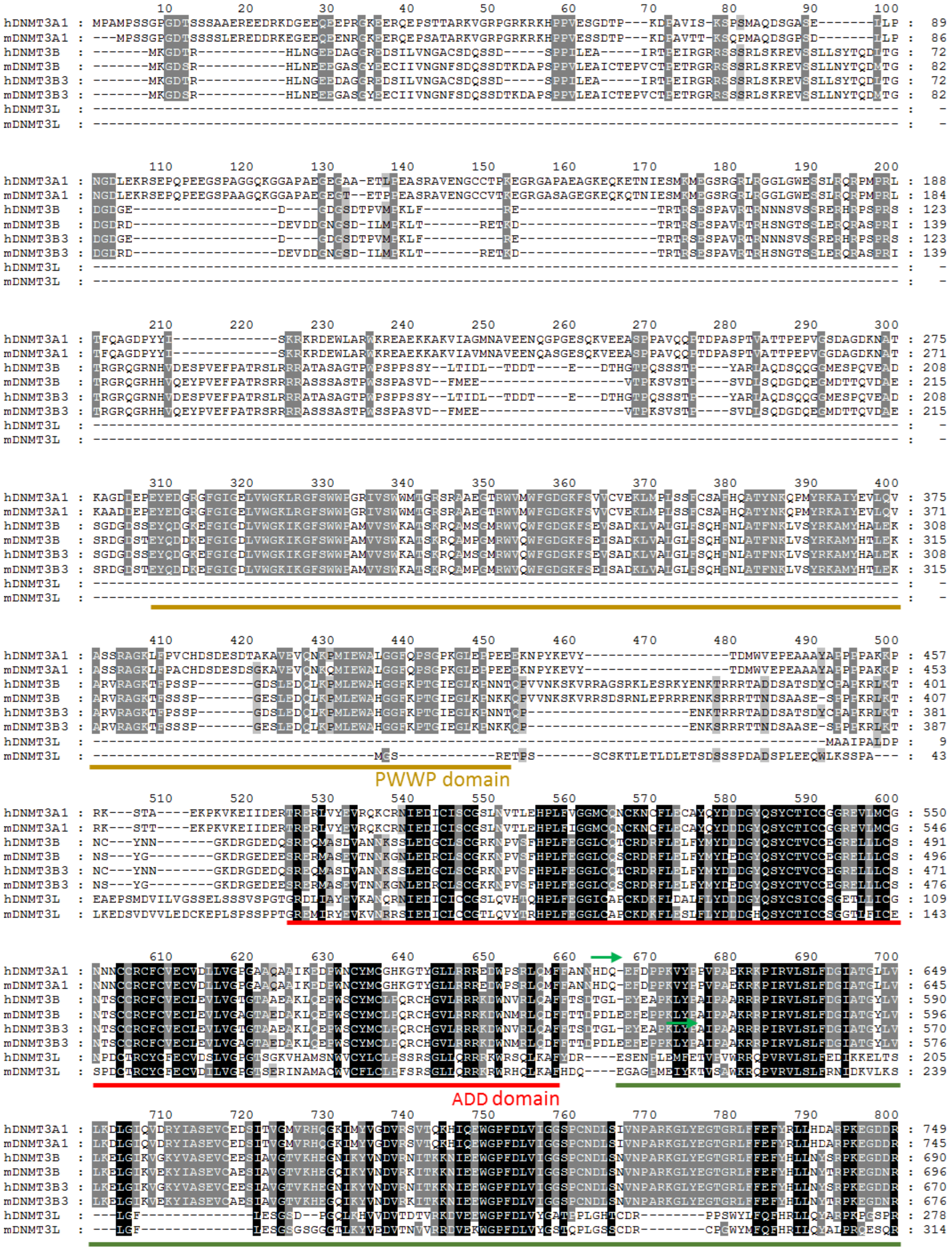

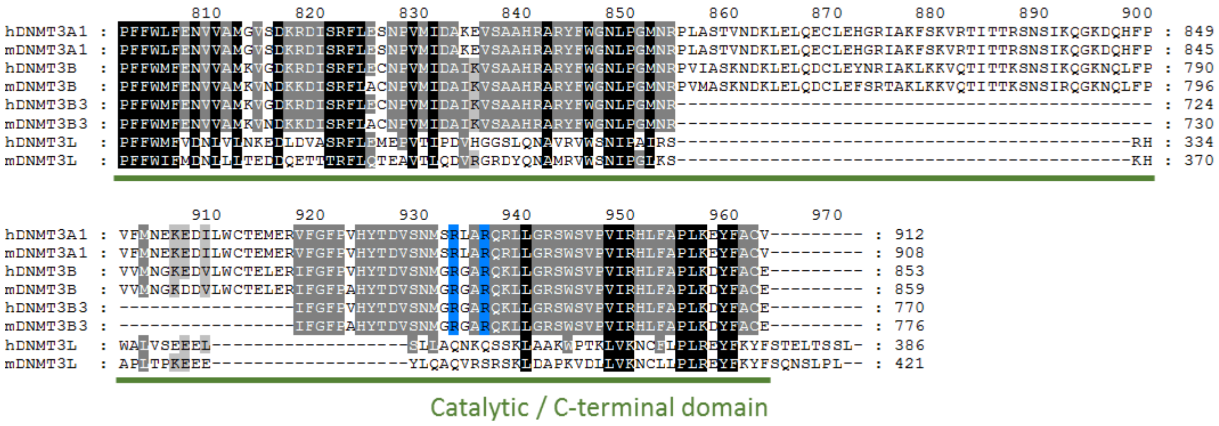

Supplemental Figure 1: Sequence alignment of human and mouse DNMT3A, DNMT3B, DNMT3B3 and DNMT3L. The first residues resolved in the DNMT3A2/3B3-nucleosome structure are labelled by green arrows. The key DNMT3B3 residues interacting with the nucleosome acidic patch (R740 and R743, highlighted in blue) are conserved between DNMT3A and DNMT3B. The sequences were retrieved from the UniProt entries: hDNMT3A1 Q9Y6K1, mDNMT3A1 O88508, hDNMT3B1 Q9UBC3, mDNMT3B1 O88509, hDNMT3B3 Q9UBC3-3, mDNMT3B3 O88509-3, hDNMT3L Q9UJW3, mDNMT3L Q9CWR8.

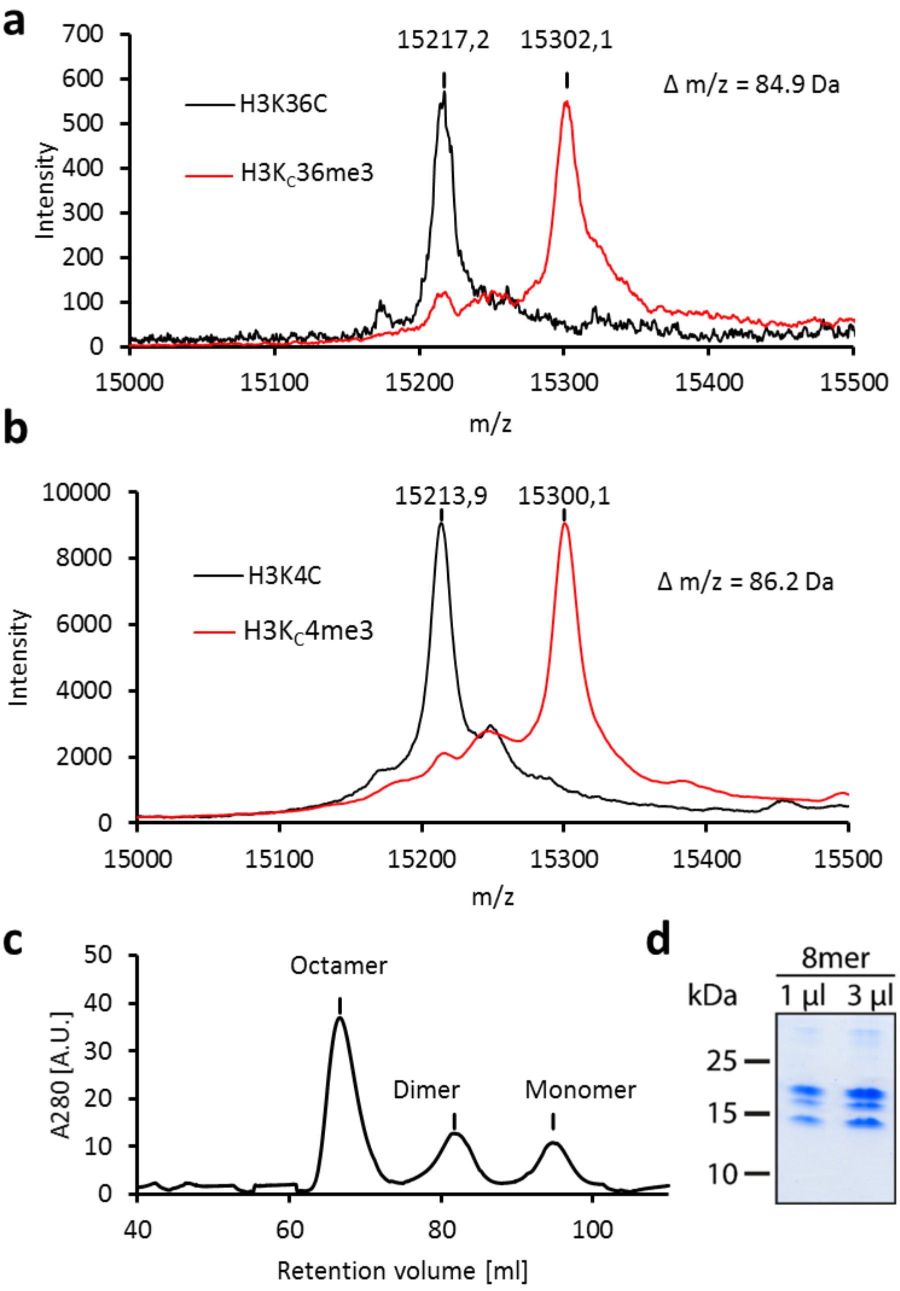

Supplemental Figure 2: Quality control of the modified histones and octamer purification. (a) MALDI mass spectrum of unconverted H3K36C and the H3K$_C$36me3 containing H3 proteins, showing the expected mass shift of 86 Da. **(b)** MALDI mass spectrum of unconverted H3K36C and the H3K$_C$4me3 containing H3 proteins, showing the expected mass shift of 86 Da. **(c)** Chromatogram of the size exclusion chromatography after histone octamer refolding, demonstrating adequate separation of the fully assembled octamer particles from partially assembled products such as dimers. **(d)** Coomassie BB-stained SDS gel of a sample taken from the octamer peak from B. All four individual histone bands are visible.

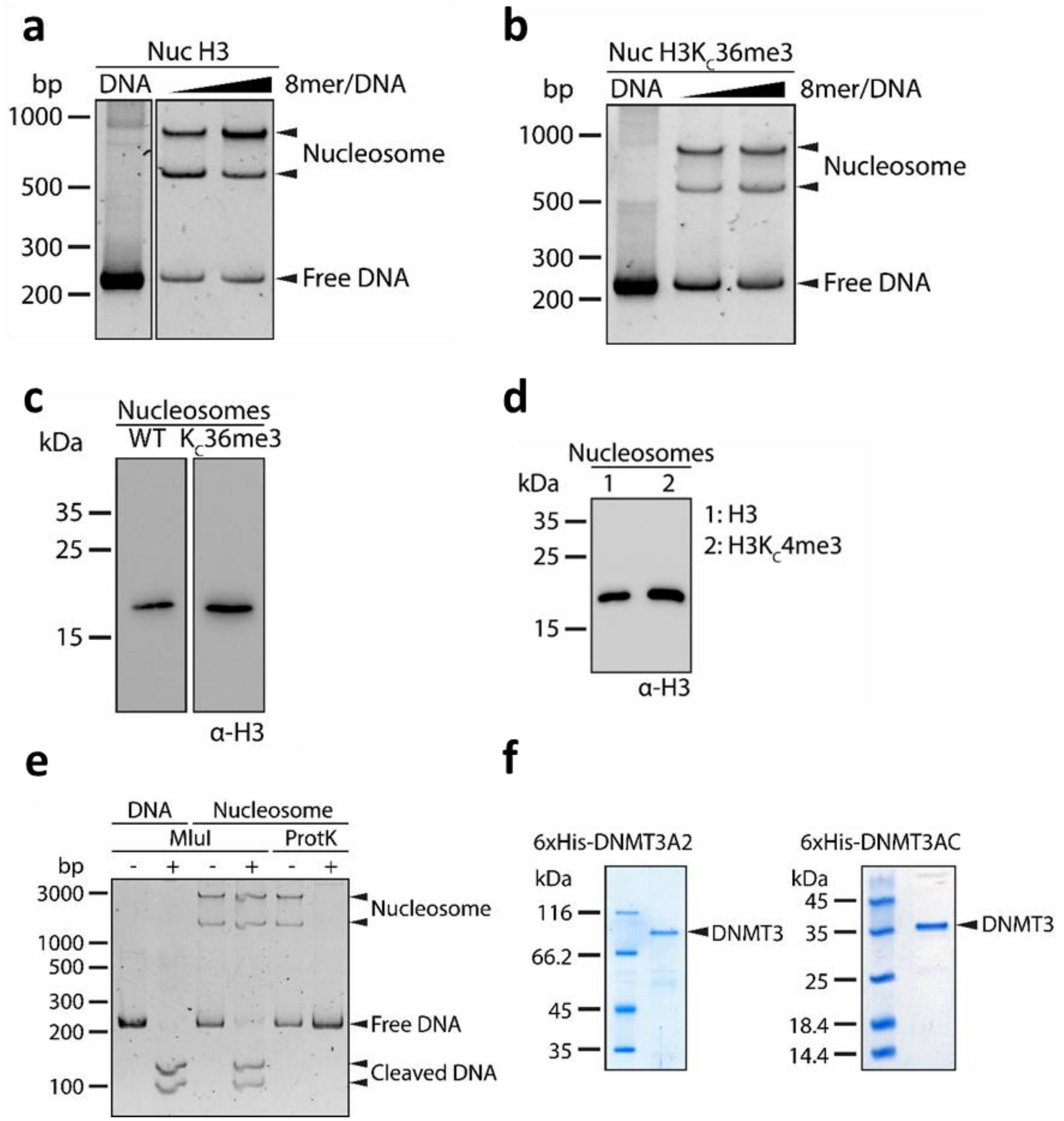

Supplemental Figure 3: Controls related to nucleosome reconstitution and methylation. (a, b) EMSA experiments showing nucleosome reconstitution for unmodified nucleosomes (a) and H3K$_C$36me3-analog containing nucleosomes (b). Indicators mark the unshifted free DNA bands at 240 bp and the shifted nucleosome bands. Two different octamer to DNA ratios were used for each reconstitution, yielding roughly similar nucleosome amounts. The images show 6% PAA gels stained with GelRed. **(c, d)** Western blot of the same nucleosome amounts using an antibody against H3 (Abcam ab1791, 1:5000). **(e)** Native PAA gel of DNA and nucleosome samples after MluI digestion, as well as of nucleosomes after proteinase K treatment. For MluI, all unbound DNA is cleaved into two fragments while the nucleosomal DNA remains intact. The image shows an 8% PAA gel stained with GelRed. **(f)** Purified DNMT3A2 and DNMT3AC proteins used in this study. The image shows Coomassie BB-stained SDS gels.

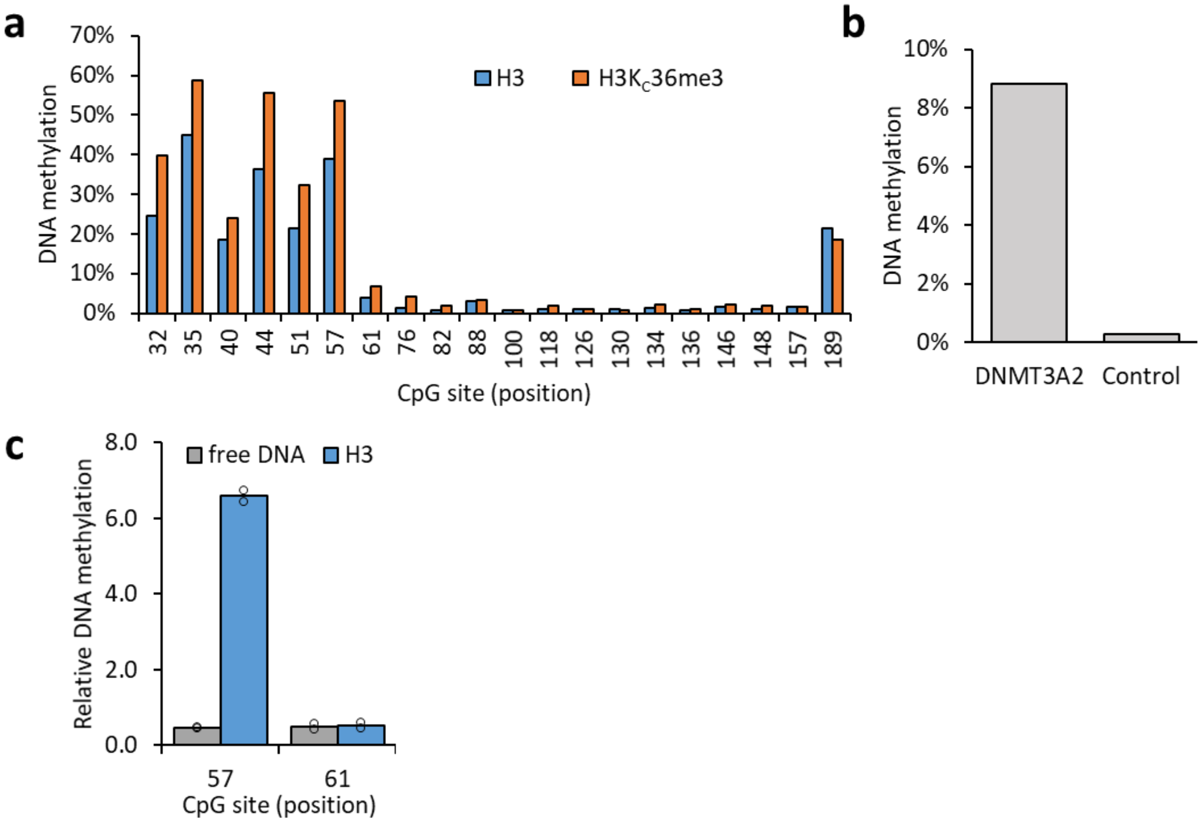

Supplemental Figure 4: Additional data related to nucleosome methylation. (a) Example of CpG site methylation levels of unmodified and H3K$_C$36me3 modified nucleosomes with DNMT3A2. **(b)** Overall methylation level of free DNA methylated by DNMT3A2 and of unmethylated free DNA used as bisulfite conversion control. **(c)** Comparison of the relative methylation levels of CpG sites 57 and 61 in the nucleosome methylation experiments and in free DNA for DNMT3AC.

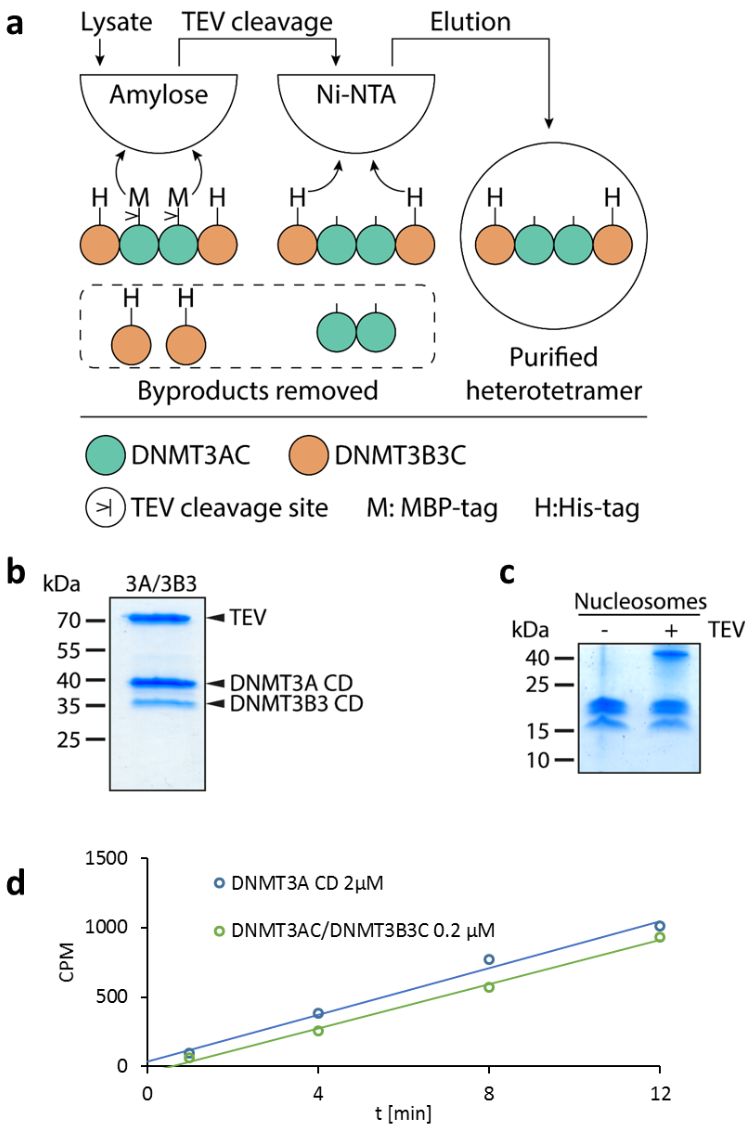

Supplemental Figure 5: Additional data related to the purification of DNMT3AC/3B3C heterotetramers. **(a)** Schematic workflow for the purification of DNMT3AC/3B3C heterotetramers. **(b)** Coomassie-stained SDS-gel of the purified DNMT3AC/3B3C heterotetramers. **(c)** TEV cleavage control of recombinant nucleosomes. **(d)** Radioactive methylation kinetics of the purified DNMT3AC/3B3C heterotetramers in comparison with DNMT3AC showing strong stimulation of the activity of DNMT3AC by DNMT3B3C.

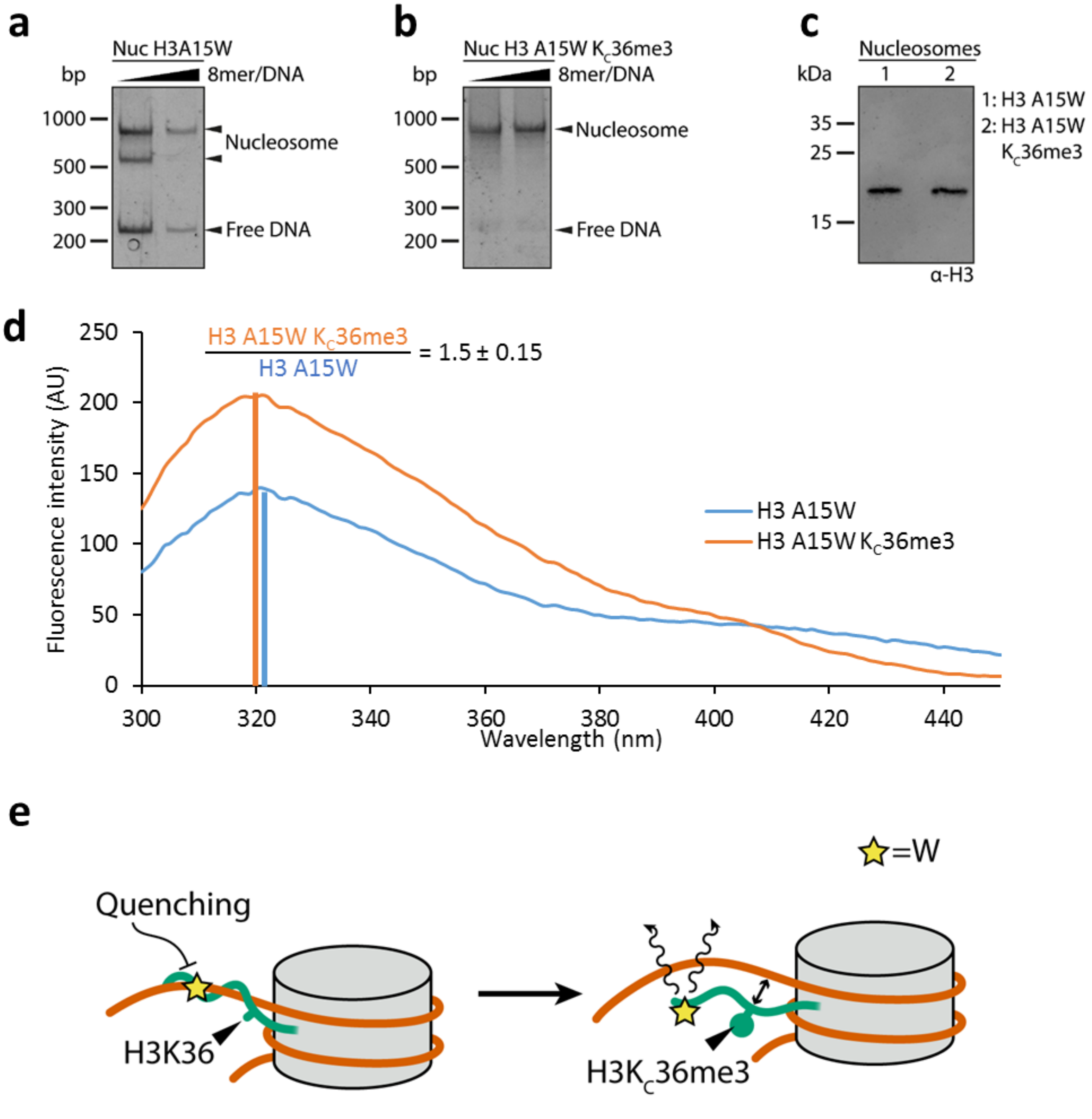

Supplemental Figure 6: Fluorescence spectroscopy experiments investigating the interaction of the H3-tail with the linker DNA. (a, b) EMSA experiments showing reconstitution for nucleosomes containing H3A15W (a) and H3A15W in combination with H3K$_C$36me3 (b). Indicators mark the unshifted free DNA bands at 240 bp and the shifted nucleosome bands. **(c)** Western blot of the same nucleosome amounts using a histone H3 antibody. **(d)** Fluorescence emission spectra of recombinant nucleosomes containing H3A15W either with or without H3K$_C$36me3. The average ratio of the fluorescence intensities of H3 A15W with and without K$_C$36me3 derived from 3 independent experimental repeats is indicated. **(e)** Schematic model of the different histone tail binding dynamics to the linker DNA with and without H3K$_C$36me3, which are leading to a difference in W15 fluorescence intensity.

Supplemental Figure 7: Uncropped versions of gel images shown in the main figures. (a) Uncropped image from Figure 3a. **(b)** Uncropped image from Figure 4c. Relevant lanes are indicated by asterisks. The marker lane is labelled by M. Marker sizes are indicated in base pairs (bp).

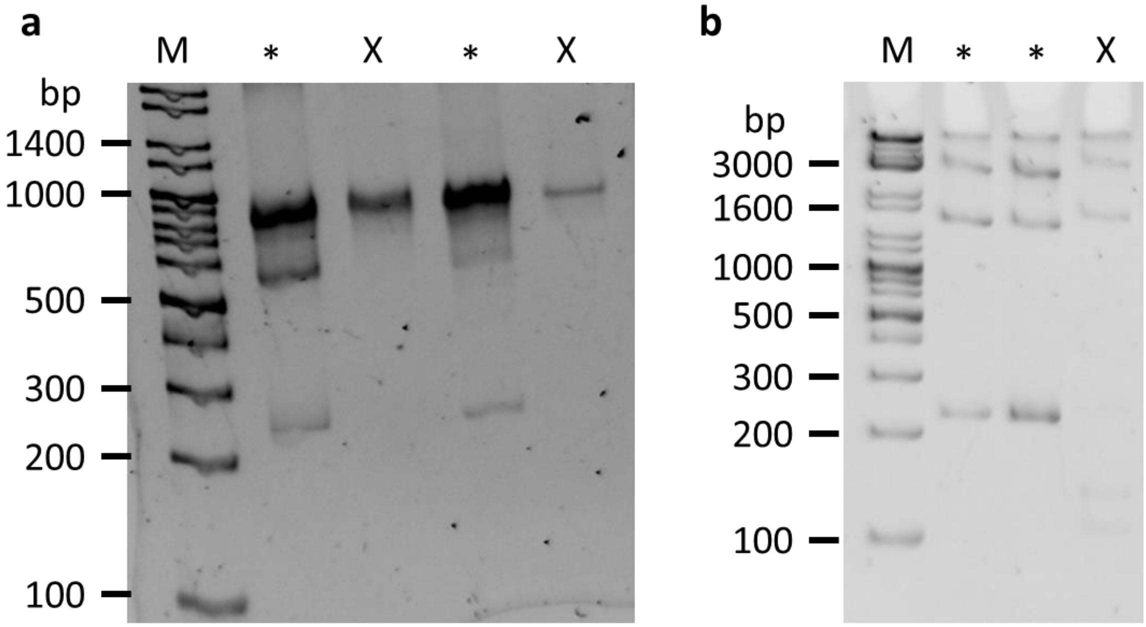

Supplemental Tables

Supplemental Table 1: List of nucleosome and DNA methylation experiments. Reads indicates the minimal number of reads for each CpG site. Filename refers to the data deposited in https://doi.org/10.18419/darus-1252.

Enzyme	Repeat	Enzyme c	Substrate	Reads*	Filename
DNMT3A2	1	0.5 µM	Nuc H3.1	1332	DNMT3A2_WT_Nuc_unmod_R1
			Nuc H3.1K36me3	61	DNMT3A2_WT_Nuc_K36me3_R1
	2	2 µM	Nuc H3.1	3147	DNMT3A2_WT_Nuc_unmod_R2
			Nuc H3.1K36me3	266	DNMT3A2_WT_Nuc_K36me3_R2
	3	1.4 µM	Nuc H3.1	1165	DNMT3A2_WT_Nuc_unmod_R3
			Nuc H3.1K36me3	1126	DNMT3A2_WT_Nuc_K36me3_R3
	4	2.8 µM	Nuc H3.1	935	DNMT3A2_WT_Nuc_unmod_R4
			Nuc H3.1K36me3	843	DNMT3A2_WT_Nuc_K36me3_R4
	5	0.5 µM	Nuc H3.1	3269	DNMT3A2_WT_Nuc_unmod_R5
			Nuc H3.1K36me3	31074	DNMT3A2_WT_Nuc_K36me3_R5
DNMT3A2	1	1.4 µM	Free DNA	2418	DNMT3A2_WT_DNA_R1
	2	2.8 µM	Free DNA	2873	DNMT3A2_WT_DNA_R2
-	1	-	Free DNA	28337	Bisulfite_Conttrol_DNA_R1
DNMT3AC	1	10 µM	Nuc H3.1	2923	DNMT3AC_Nuc_unmod_R1
			Nuc H3.1K36me3	14190	DNMT3AC_Nuc_K36me3_R1
	2	5 µM	Nuc H3.1	4385	DNMT3AC_Nuc_unmod_R2
			Nuc H3.1K36me3	24846	DNMT3AC_Nuc_K36me3_R2
DNMT3A2	1	0.8 µM	Nuc H3.1	72365	DNMT3A2_Nuc_unmod_R1x
			Nuc H3.1K4me3	195290	DNMT3A2_Nuc_K4me3_R1x
	2	2.5 µM	Nuc H3.1	9964	DNMT3A2_Nuc_unmod_R1x
			Nuc H3.1K4me3	53051	DNMT3A2_Nuc_K4me3_R1x
	3	4.9 µM	Nuc H3.1	28096	DNMT3A2_Nuc_unmod_R1x
			Nuc H3.1K4me3	101067	DNMT3A2_Nuc_K4me3_R1x
DNMT3AC/ DNMT3B3C	1	525 nM	Nuc H3.1	53709	DNMT3AC_DNMT3B3C_4mer_Nuc_unmod_R1x
	2	175 nM	Nuc H3.1	320287	DNMT3AC_DNMT3B3C_4mer_Nuc_unmod_R2x
	3	175 nM	Nuc H3.1	631362	DNMT3AC_DNMT3B3C_4mer_Nuc_unmod_R3x
	4	70 nM	Nuc H3.1	860098	DNMT3AC_DNMT3B3C_4mer_Nuc_unmod_R4x
	5	35 nM	Nuc H3.1	760095	DNMT3AC_DNMT3B3C_4mer_Nuc_unmod_R5x
