# Supplementary material for: Methylation of recombinant mononucleosomes by DNMT3A demonstrates efficient linker DNA methylation and a role of H3K36me3
Source: Commun Biol. 2022 Mar 2;5:192. doi: 10.1038/s42003-022-03119-z (PMC8891314; doi:10.1038/s42003-022-03119-z)
Supplement: Supplementary file 3 — Description of Additional Supplementary Files [file 42003_2022_3119_MOESM3_ESM.pdf]

Description of Additional Supplementary Files

File name: Supplemental Data 1
Description: Source data.
